# Cross-training needs among community-based clinicians in HIV and substance use

**DOI:** 10.1186/s12909-022-03682-3

**Published:** 2022-08-19

**Authors:** Kasey Claborn, Kelli Scott, Sara J. Becker

**Affiliations:** 1 Department of Psychiatry, University of Texas at Austin Dell Medical School, 1501 Red River St, Austin, TX 78712 USA; 2 Center for Alcohol and Addiction Studies, Brown University School of Public Health, 121 S. Main Street, Providence, RI 02903 USA; 3 Northwestern University Feinberg School of Medicine, 663 N St. Clair Street, Chicago, IL 60611 USA

**Keywords:** HIV, Substance use disorder, PrEP, Treatment, Training, Clinician

## Abstract

**Background:**

People with double burden of HIV and substance use have poorer treatment engagement and worse treatment outcomes. Cross-training of HIV and substance use disorder clinicians can potentially enhance the scale up and quality of integrated care. Research is needed on clinicians’ areas of greatest training need in order to inform training development.

**Methods:**

Data from semi-structured individual interviews with 16 HIV and 13 substance use disorder clinicians (*N* = 29) examining clinician perspectives on their training needs were analyzed using thematic analysis focused on both a priori and emergent subthemes.

**Results:**

Several key emergent subthemes were identified across the a priori themes of: 1) past training experiences; 2) gaps in training; and 3) training and supervision format/structure. Both HIV and substance use clinicians reported they had received minimal formal cross-training and had mostly been trained “on the job.” Clinicians also emphasized gaps in training regarding sensitivity and anti-stigma, the latest medications for opioid use disorder, and HIV prevention/treatment and referral resources. Regarding training and supervision format, clinicians cited didactic workshops and ongoing supervision as appealing strategies.

**Conclusions:**

Results show that lack of formal and updated training for clinicians is an important gap in providing integrated HIV and substance use treatment. Didactic workshops and ongoing support strategies that address stigma, medications for HIV and substance use disorder, and referral resources are likely to be particularly valuable.

**Supplementary Information:**

The online version contains supplementary material available at 10.1186/s12909-022-03682-3.

The Centers for Disease Control and Prevention estimates that 1.2 million U.S. adolescents and adults (aged 13 and older) are living with HIV, with 13.3% of individuals undiagnosed [1]. Alcohol and drug use are common among people living with HIV (PLWH). More than 80% of PLWH report a history of drug use and nearly one in four meet diagnostic criteria for a substance use disorder [2]. Substance use is associated with both reduced initiation and retention on anti-retroviral therapy for PLWH, thus increasing the likelihood of further transmission and reduced survival for this population [3, 4]. PLWH who use drugs are also at elevated risk of an array of negative outcomes including medical (e.g., hepatitis C, tuberculosis) and psychiatric (e.g., depression, anxiety) comorbidity, neurocognitive impairment [5], drug overdose [6], and increased mortality [7–9]. These negative outcomes are often exacerbated by inadequate access to medical care [10] which is partly driven by siloed health systems in which HIV and substance use care are delivered separately [11], and patient-level barriers such as unstable motivation to change and negative experiences with healthcare providers [12]. People who use drugs report experiencing dehumanization and discrimination in healthcare settings as a result of drug use which results in delaying initiation of services, not disclosing drug use to their providers, downplaying pain, and seeking healthcare in community-based organizations instead of large institutional settings as these organizations tend to be non-stigmatizing, more accepting, and prioritize mutual respect and connection [13].

Integrated care, using an inter-disciplinary, team-based, coordinated approach, has been identified as the ideal treatment for PLWH who use drugs [14]. Recently, the U.S. White House released the National HIV/AIDS Strategy for 2022–2025 which included a specific goal for improving integrated care for substance use services that focuses on early intervention, HIV testing, and provision of PrEP and antiretroviral therapy [15]. The National Academies of Science, Engineering, and Medicine, the Infectious Disease Society of America, and the HIV Medicine Association also recommend integrated HIV and substance use disorder care, including medication for opioid use disorder and harm reduction programs [16, 17]. This guidance also recommends the implementation of multi-level strategies focused on reducing stigma in treatment settings for PLWH who use drugs [16]. The myriad benefits of service integration include improved tracking and monitoring of patients, enhanced coordination of comprehensive treatment plans, decreased healthcare costs, and increased patient outcomes. Despite established guidelines, multiple barriers prevent the integration of HIV and substance use care. One key organizational barrier is insufficient screening and assessment of substance use disorders in HIV care clinics and insufficient screening and assessment of HIV in substance use treatment settings [4, 18, 19].

Formal cross-training in screening and evidence-based treatments for HIV and substance use disorder could enhance the quality of integrated care across treatment settings. Montague et al. (2015) surveyed 119 HIV and 159 addiction treatment clinicians and found that clinicians agreed with statements indicating that they needed more knowledge and skills in effective assessment and identification for both diseases [20]. HIV providers received less formal training about substance use compared to addiction treatment clinicians. However, both HIV and substance use disorder clinicians did not view formal training in substance use and HIV, respectively, as a priority. This lack of prioritization of additional training may be due to a variety of factors, including perceptions that such training is outside of their scope of practice, beliefs that patients will not be receptive to HIV/substance use intervention, or presence of numerous competing demands on provider time [20].

Although integrated care guidelines exist for HIV and substance use disorders, there is a lack of research exploring the specific training gaps and potential training structure and content that would be best received by community clinicians providing substance use and HIV treatment. This research is particularly important in light of findings suggesting that clinicians may not prioritize training in integrated intervention [20]. The current qualitative data analysis expands on prior work by examining prior training experiences, the perceived need for cross-training and preferred training structure/format among both HIV and substance use disorder clinicians. Findings from this study may inform the format and content of new cross-training curricula to enhance integrated care provision for PLWH who use drugs in line with new treatment guidelines.

## Methods

### Parent study

The current study was embedded within a larger program of research investigating clinicians’ perspectives on facilitators and barriers to HIV and substance use disorder care [21]. The parent study conducted qualitative interviews with clinicians to assess their experiences working with PLWH who use drugs and their strategies to improve retention in care. Data collection for the parent study was grounded in syndemics theory considering the interaction of HIV and substance use disorder interact synergistically to worsen health outcomes for dually diagnosed patients [22]. The current analysis specifically focused on clinician impressions of training needs, prior training experiences, and training preferences.

### Participants

Twenty-nine clinicians, 16 HIV and 13 substance use, were recruited in the New England area via email in 2015 [21]. Inclusion criteria for participants included: (a) minimum of 18 years-old; (b) currently employed at an HIV or substance use disorder treatment clinic for a minimum of 6 months; (c) minimum of 1 year experience working with people living with HIV (PLWH) or patients at-risk for HIV who also have a co-morbid substance use disorder; and (d) held one of the following position titles: counselor, case manager, medical liaison, medical resident, nurse practitioner, nurse, outreach worker, physician, or social worker. Exclusion criteria included being unable to speak English or provide written consent. All staff who contacted the research team and met the inclusion criteria agreed to participate in the interviews.

### Procedure

Study procedures were approved by the University of Texas at Austin Institutional Review Board. Qualitative data collection and analysis followed the consolidated criteria for reporting qualitative research (CORE-Q) [23]. The research team developed a semi-structured interview guide to assess clinician perceptions of treatment needs among PLWH who use drugs (see Supplemental Material for the full interview guide). A female interviewer with a Ph.D. in Clinical Psychology and extensive training in qualitative research methods conducted face-to-face, in-depth qualitative interviews with both HIV and substance use disorder treatment clinicians. Qualitative interviews were audio-recorded. The interview was not known to participants prior to the interview session and interviews were conducted privately and in one session at the participants’ agency (either a community-based HIV or substance use treatment clinic). Interviews ranged in duration from 45 to 90 minutes. Participants provided written informed consent prior to participation and completed a short demographic survey. Each participant was compensated $50 for participation. This study is a secondary analysis of emergent qualitative data focused on three a priori themes: (a) clinicians past cross-training experience; (b) gaps in clinicians’ current cross-training in HIV and substance use treatment; and (c) clinicians’ preferences for the format/structure of cross-training and ongoing supervision. Interview transcripts were not returned to participants for review due to study time constraints and to reduce burden placed on participating clinicians.

### Data analysis

After each interview, a debriefing and data summary was performed by the interviewer and either the study Principal Investigator or a Co-Investigator with qualitative research expertise. All digital recordings were transcribed verbatim into a Word document and entered into NVivo qualitative data management software for organization and coding of qualitative data. Applied thematic analysis, a rigorous, deductive approach, was completed to identify and report common themes within the data [24]. Coding occurred in two major stages. In Stage 1, the following analytical steps were utilized to develop an initial coding structure and identify quotes associated with the major themes: a primary code book containing a comprehensive list of major a priori themes was developed based upon the interview guide; two PhD level researchers trained in qualitative analysis coded all transcripts using only the a priori themes; and coders met weekly to review assignments, identify areas of agreement, and resolve discrepancies until 100% consensus was obtained.

Stage 2 was initiated after the coding structure was finalized. In this stage, all of the quotes pertaining to training needs, experiences, and preferences were reviewed. Data mining tools in NVivo were applied to ensure that all relevant passages were included, using targeted queries of the data via terms such as “training,” “supervision,” and “learn.” Two different independent coders independently reviewed all of the quotes and assigned them to emergent sub-themes. The independent coders met periodically to review all assigned codes until agreement was reached about all relevant emergent themes and to achieve theme saturation. Findings were not shared with participants following completion of data analysis.

## Results

### Sample characteristics

Study participants (*N* = 29) were predominantly female (72%), and between the ages of 45 to 54 (34%). The majority of participants had a college degree (31%), Master’s degree (21%), or a doctorate (24%). Participants also represented a range of positions, including medical assistants (3%), nurses (28%), case managers (4%), social workers (4%), counselors (21%), clinical supervisors (14%), physician assistants (3%), medical fellows (7%), and attending physicians (17%). This sample was also highly experienced in their field, with nearly 40% having 15 or more years of experience in either HIV or substance use disorder. See Table 1 for participant characteristics.

**Table 1:** Participant demographics (*N* = 29)

	Employed in HIV care (***n*** = 16)	Employed in SU care (***n*** = 13)	Total (***n*** = 29)
	***N (%)***	***N (%)***	***N (%)***
Gender Identity			
Male	2 (7%)	5 (17%)	7 (24%)
Female	14 (48%)	7 (24%)	21 (72%)
Gender Queer	0 (0%)	1 (3%)	1 (4%)
Age			
18–24	0 (0%)	1 (3%)	1 (4%)
25–34	2 (7%)	6 (21%)	8 (28%)
35–44	4 (14%)	1 (3%)	5 (17%)
45–54	7 (24%)	3 (10%)	10 (34%)
55+	3 (10%)	2 (7%)	5 (17%)
Education			
Some College	1 (3%)	3 (10%)	4 (14%)
Licensed Practical Nurse	3 (10%)	0 (0%)	3 (10%)
College Graduate	3 (10%)	6 (21%)	9 (31%)
Master’s Degree	2 (7%)	4 (14%)	6 (21%)
Doctorate	7 (24%)	0 (0%)	7 (24%)
Position Title			
Medical Assistant	1 (3%)	0 (0%)	1 (3%)
Nurse (LPN and RN)	6 (21%)	2 (7%)	8 (28%)
Case Manager	0 (0%)	1 (3%)	1 (4%)
Social Worker	1 (3%)	0 (0%)	1 (4%)
Counselor	0 (0%)	6 (21%)	6 (21%)
Clinical Supervisor	0 (0%)	4 (14%)	4 (14%)
Physician Assistant	1 (3%)	0 (0%)	1 (3%)
Fellow	2 (7%)	0 (0%)	2 (7%)
Attending Physician	5 (17%)	0 (0%)	5 (17%)
Experience working with PWUD (in years)			
0–1	0 (0%)	0 (0%)	0 (0%)
2–5	2 (7%)	3 (10%)	5 (17%)
6–10	3 (10%)	6 (21%)	9 (31%)
11–15	3 (10%)	1 (3%)	4 (14%)
> 15	8 (28%)	3 (10%)	11 (38%)
Experience working with PLWH (in years)			
0–1	1 (3%)	0 (0%)	1 (4%)
2–5	2 (7%)	4 (14%)	6 (21%)
6–10	3 (10%)	5 (17%)	8 (28%)
11–15	2 (7%)	0 (0%)	2 (7%)
> 15	8 (28%)	4 (14%)	14 (21%)

*SU* substance use, *PWUD* people who use drugs, *PLWH* people living with HIV, *LPN* licensed practical nurse, *RN* registered nurse

### Emergent themes on cross-training in HIV and substance use among clinicians

Emergent data revealed an array of sub-themes in response to the 3 a priori themes: (a) prior training experiences, (b) needs for HIV and substance use disorder cross training; and (c) preferred training structure/format. Table 2 presents a definition of each theme, example emergent sub-themes, and illustrative quotes.

**Table 2:** List of a priori themes, emergent themes, definitions, and exemplar quotes

A Priori Themes	Emergent Themes	Definition	Exemplar Quote
Past Training Experiences	On the Job Training	Past training experiences in substance use or HIV being primarily “on the job”	HIV clinician: “*The training I’ve had has really been just experiential.”*
	Coursework/Workshops	Past training experiences in substance use or HIV being from many years ago	Substance use disorder clinician: “*...but also like while I was in college, we had the section on HIV.”*
Gaps in Training	Substance Use Treatment	Clinicians not having enough training in the assessment or treatment of substance use disorders.	HIV clinician: *“… Realizing when it’s appropriate and not appropriate to prescribe opiates. I don’t think most of us get a lot of specific training in substance abuse issues.”*
	HIV Prevention and Treatment	Clinicians not having enough training in the prevention or treatment of HIV.	Substance use disorder clinician: “*Maybe more what’s been happening with HIV? We have answers. We don’t hear about it as much as we used to. How does it go with methadone? Just a little more education. I mean, I just know what I’ve been learning, but it’s not a whole lot.”*
	Sensitivity Training	Need for training to reduce stigma or judgment surrounding substance use and/or HIV.	HIV clinician: *“I think that it is probably helpful for all providers to have a real understanding of substance abuse. I think that there are still providers out there who don’t think of it from a medical or a disease like any other, there’s still, even for providers, a lot of stigma. We hear that all the time from patients. “I like coming here, because you guys don’t judge me.”*
	Referral Resources	Need for training in referral resources for substance use and/or HIV.	Substance use disorder clinician: *“…I feel like I don’t always know where to send them [clients] for HIV..”*
	Mental Health Treatment	Gaps in training related to mental health assessment and treatment.	HIV clinician: *“I don’t know where the line is when it’s active suicidality where you have the right to lock him up so he isn’t a danger to himself,* versus *passive suicidality.”*
Training and Supervision Format/Structure	Workshop Training	Preference for workshop training.	Substance use disorder clinician: *“…think it depends upon each person’s learning style, but I think, hypothetically speaking, if [doctor] were to give a workshop or a day training, or even a half a day training, you would get so much more outta that than reading a PowerPoint”*
	Training Frequency	Preference for training to be held regularly	Substance use disorder clinician: *“I don’t know if it’s just site-to-site or if it’s something that should come from the Department of Health as a requirement cuz you work in this kinda setting…there should be more of an emphasis placed on updating people who are working in the fields with these particular* *clients, whether it’s once a year, at a minimum.”*
	Resources/ Referrals Incentives	Desire for information about referral options and community resources Desire for incentives to attend training such as continuing education credits or lunch.	HIV clinician: *“Probably just even a basic… resource list that gets updated on a regular basis.”* Substance use disorder clinician: *“Offer CEUs. That’s how you get us to come.”* HIV clinician: *“People will come when they have food.”*
	Supervision	Preference for receiving ongoing supervision.	Substance use disorder clinician: *“Someone who might be maybe passionate about the material, the information, and someone* *who has worked directly with the population I work with and has an understanding of maybe both where we’re coming from, as well as everything they know about the medical side. Yeah. Weekly, with my supervisor, sit down and talk about each case, and supervision can happen at any given point in time.”*

#### Past training experiences

HIV and substance use disorder clinicians both reported that they had minimal formal cross-training in substance use and HIV treatment, respectively. Five HIV clinicians (31%) reported that their past training in substance use disorder treatment had occurred primarily “on the job,” while others reported that they received brief training in substance use disorder treatment “ages ago,” most commonly during undergraduate or graduate training.

Substance use disorder clinicians provided similar feedback. Three clinicians reported that they had “very minimal” or “no training” in HIV, whereas six reported that they had received some specific HIV training via workshops and/or coursework. Of those substance use clinicians who reported receiving specific HIV training, two noted that it was mandated by their agency and one noted that the mandated training was not beneficial because “it’s just monotonous” (1027). Both HIV and substance use clinicians consistently noted that cross-training would be beneficial to their practice.

#### Gaps in training

When asked to consider specific areas of training need, the clinicians highlighted multiple gaps in their training. One of the most frequently mentioned training gaps, cited by nearly 25% (*N* = 7) of participants (both HIV and substance use clinicians) was training in understanding, assessing, and treating substance use disorders. For instance, one HIV clinician noted, *“I think that it is probably helpful for all providers to have a real understanding of substance abuse. I think that there are still providers out there who don’t think of it from a medical or a disease like any other”* (1006). Four out of the seven providers requesting training in substance use treatment specifically wanted information on medication for opioid use disorder (MOUD). A substance use disorder clinician said, *“At this day and age, I think all prescribers should have some form of methadone understanding, training on methadone and Suboxone, just with the amount of use over that”* (1027).

Another training gap noted by seven respondents (all substance use clinicians) was information related to HIV prevention and treatment. Substance use clinicians reported a need for training in a wide range of HIV topics, including general HIV knowledge, new medications for HIV prevention and treatment (including Pre-Exposure Prophylaxis, i.e. PrEP), impact of HIV on methadone treatment, impact of alcohol on HIV, and comorbidity between HIV and Hepatitis C. One substance use disorder clinician indicated that*: “We’ve all heard of the new medication, but nobody knows what it is. I don’t, other than what I’ve gathered from my patients. Nobody’s told me specifically what it does, how it works”* (1017).

The next most popular sub-themes were sensitivity training and referral resources, with each suggested by six respondents. Both HIV and substance use clinicians expressed a need for sensitivity or anti-stigma training, with HIV clinicians (*n* = 3) and substance use (*n* = 3) clinicians noting the prominence of judgmental views towards PLWH who use drugs. One HIV clinician noted: *“patients first and foremost want providers that are nonjudgmental”* (1015), while a substance use clinician noted that providers need training because patients *“have been abused by doctors in facial expressions and words”* (1018).

Referral resources were also a common training need, highlighted by both HIV (*n* = 4) and substance use (*n* = 2) clinicians. One HIV clinician indicated that*: “Resources, I think, is always a good thing to learn what’s available [for substance use disorder treatment] because what’s available in one city is not available in another city”* (1008). Relatedly, several clinicians asked for explicit training about levels of care to guide their referrals.

Finally, two HIV clinicians expressed a desire for training in mental health assessment and treatment, including how to accurately assess mental health symptoms and suicide risk in their population. One of the HIV clinicians indicated that*: “I don’t think we’re properly equipped or that we haven’t been trained in a way to very much prioritize mental health....” (1014).*

#### Training and supervision format/structure

Clinicians were also asked about their preferences for receiving training in the future, including training format, length, and frequency. Clinicians, and especially substance use disorder clinicians, most frequently endorsed workshop training as their preferred training format (*n* = 1 HIV clinician, *n* = 8 substance use disorder clinicians). They specifically indicated a desire for an in-person workshop training held on a regular basis (e.g., every 6 months or yearly) with an experienced instructor. Clinicians noted they ideally wanted support resources such as packets or pamphlets with HIV or substance use information. One substance use disorder clinician described their ideal training:

*“I think a training where maybe someone outside of the agency is—that professionally knows those two diseases [HIV and Hepatitis C] and how they progress and what they’re doing about them, what’s happening, what’s current with them, that’d be huge...think if it was a training that was interesting and upbeat and allow us to ask lots of questions and focus on current and how it directly impacts our patients, I think that’d gather a lot more interest, a lot more interaction.”* (1029).

Several clinicians further noted that due to the time demands in their jobs, it would be important to provide incentives for their attendance (e.g., lunch, continuing education credits, or overtime pay).

When discussing ongoing training needs, supervision emerged as a key sub-theme. Of note, this sub-theme did not emerge as a topic of discussion among HIV clinicians, whereas five of the 13 substance use disorder clinicians (38%) shared their perspective that weekly or as needed supervision could support their training in HIV prevention and intervention.

## Discussion

To our knowledge, this is the first qualitative study to examine the cross-training needs of HIV and substance use disorder treatment clinicians. In general, both HIV and substance use clinicians desired additional cross-training in assessment and treatment. Although many of the clinicians reported some form of previous training on these topics, they noted that this training was provided a long time ago (e.g. in college or graduate training), occurred primarily “on the job” without formal instruction, and/or was monotonous. These findings about the low rates of cross-training are consistent with prior research documenting a need for more addiction-related curriculum, using interactive teaching methods, to be offered throughout medical training [25]. In recent years, some medical programs have begun to integrate buprenorphine waiver training into their curricula [26, 27], but the interplay of HIV and substance use risk still remains an area in need of focus.

In regards to areas of specific training need, both HIV and substance use clinicians discussed a need for sensitivity or anti-stigma training, as well as training in the latest medications and referral resources. In line with previous studies, some clinicians expressed that stigmatized behavior towards PLWH and people who use drugs could serve as a barrier to treatment retention [13, 28–30]. Medications to treat opioid use disorder and medications to prevent/treat HIV were also cited as areas in need of further training. These findings are consistent with prior research indicating that insufficient knowledge of HIV, PrEP, and substance use disorder treatment options hinders linkage to appropriate treatment and integrated care for patients with comorbid HIV and substance use disorder [31].

The current results underscore the need for more formal cross-training opportunities in HIV and substance use. However, these trainings need to be carefully developed given that knowledge/skill gain in these topics may be a low priority for some practicing clinicians [20]. First, workforce training in integrated HIV and substance use care should begin in graduate training programs (e.g. in medical school, licensed substance use counseling programs) to build early knowledge about treatment best practice guidelines. Early training may be especially important, as clinicians face numerous barriers such as inability to bill for training time, high workplace productivity requirements, and low training availability once they begin practicing [16]. Second, training efforts should evaluate and prioritize areas of need, including screening and referral to treatment, identification of local HIV and substance use referral resources, and medications such as methadone and buprenorphine for opioid use disorder and PrEP and antiretroviral therapy for HIV [16]. Finally, both early and ongoing trainings should explicitly focus on stigma, and especially the intersectional stigma that may be experienced in healthcare settings by PLWH who use drugs [32, 33].

Our results also highlighted clinician preferences for training structure/format, including didactic workshops and ongoing supervision. Substance use disorder clinicians indicated a desire for an in-person workshop training far more frequently than HIV clinicians, suggesting that the ideal training strategies will likely differ by discipline. In general, clinicians expressed a desire for frequent HIV or substance use training delivered by skilled trainers. Training programs for substance use and HIV clinicians may need to incorporate strategies beyond traditional workshop training, as workshops are insufficient to promote behavior change [34] and were less preferred by HIV clinicians. Strategies such as incorporating active learning and behavioral rehearsal in training [35], and ongoing support activities such as supervision and performance feedback may be effective to enhance integrated care guideline uptake [36, 37]. Academic detailing strategies, or the provision of quick, personalized, one-on-one training in the clinician’s office [38], may also be particularly useful as it aligns with clinicians’ report of commonly learning “on the job.” Finally, all training and ongoing support strategies should incorporate intersectional stigma as a theme, including provision of stigma education, engagement with people with lived experience, and active learning opportunities to integrate destigmatizing practices (e.g. person first language) [32].

### Study limitations

The current findings must be considered within the context of several limitations. First, generalizability of the sample is limited by the focus on clinicians from New England, the majority of which were well educated with limited racial/ethnic diversity. It cannot be assumed that these results will be applicable to other regions. Second, this study was designed to focus on the provider perspective and, as such, did not consider the patient perspective. It is possible that patients would have different impressions of the areas of greatest training need in HIV and substance use treatment settings, and may offer unique views of intersectional stigma in healthcare. Finally, clinician feedback about their areas of greatest training need was likely limited by their familiarity with the discipline. Clinicians may have other areas of training need that did not emerge in these interviews due to limited provider awareness.

## Conclusion

Notwithstanding these limitations, the current study is an important first step toward understanding training needs, both with regard to topics and structure, for both HIV and substance use clinicians who serve PLWH who use drugs. Our results highlight the need for cross-training in HIV and substance use disorder among community clinicians to improve linkage to and retention in care for highly vulnerable patient populations.

In summary, cross-training in HIV and substance use disorder treatment would likely benefit from pairing initial didactics containing active learning strategies (e.g., behavioral rehearsal, role plays) with ongoing support (e.g., supervision, feedback): incentives to promote attendance would be highly desirable. Training content should include: intersectional stigma, medication for opioid use disorders and HIV, basic mental health assessment strategies, and referral resources. Delivering training using the aforementioned strategies and content areas recommended by community HIV and substance use clinicians could potentially represent a meaningful way to promote the provision of integrated care.

## Supplementary Information

**Additional file 1.** HIV Provider Individual Interview Guide.

## Data Availability

The datasets generated and/or analyzed during the current study are not publicly available due to the data containing information that could compromise research participant consent. Data are available from the first author of this manuscript on reasonable request.

## Abbreviations

PLWH: People living with HIV
PrEP: Pre-Exposure Prophylaxis

## References

[1] CDC. HIV Surveillance Report: Diagnoses of HIV Infection in the United States and Dependent Areas 2019;32. https://www.cdc.gov/hiv/library/reports/hiv-surveillance/vol-32/index.html

[2] Substance Abuse and Mental Health Services Administration. Center for Behavioral Health Statistics and Quality. Results from the 2010 National Survey on Drug Use and Health: Summary of National Findings 2011.

[3] Gonzalez A, Barinas J, O’Cleirigh C (2011). Substance use: impact on adherence and HIV medical treatment. Curr HIV/AIDS Rep.

[4] Das S, Muhetaer K, Alvarado HA. Changes in integrated HIV care in substance use treatment facilities (2015–2020) (the CBHSQ spotlight). Rockville; 2022. https://www.samhsa.gov/data/report/changes-integrated-hiv-su-facilities

[5] Weber E, Morgan EE, Iudicello JE, Blackstone K, Grant I, Ellis RJ (2013). Substance use is a risk factor for neurocognitive deficits and neuropsychiatric distress in acute and early HIV infection. J Neuro-Oncol.

[6] Wang C, Vlahov D, Galai N, Cole SR, Bareta J, Pollini R (2005). The effect of HIV infection on overdose mortality. AIDS.

[7] Altice FL, Kamarulzaman A, Soriano VV, Schechter M, Friedland GH (2010). Treatment of medical, psychiatric, and substance-use comorbidities in people infected with HIV who use drugs. Lancet.

[8] Chen IM, Huang CLC, Yeh BJ, Chien YL (2015). Health service utilization of heroin abusers: a retrospective cohort study. Addict Behav.

[9] Onyeka IN, Beynon CM, Ronkainen K, Tiihonen J, Föhr J, Kuikanmäki O (2015). Hospitalization in a cohort seeking treatment for illicit drug use in Finland. J Subst Abus Treat.

[10] Andersen R, Bozzette S, Shapiro M, St Clair P, Morton S, Crystal S (2000). Access of vulnerable groups to antiretroviral therapy among persons in care for HIV disease in the United States. HCSUS consortium. HIV cost and services utilization study. Health Serv Res.

[11] Cunningham CO, Sohler NL, Cooperman NA, Berg KM, Litwin AH, Arnsten JH (2011). Strategies to improve access to and utilization of health care services and adherence to antiretroviral therapy among HIV-infected drug users. Subst Use Misuse.

[12] Conway FN, Rountree MA, Jones KV (2021). Serving the co-morbid mental health and substance use needs of people with HIV. Community Ment Health J.

[13] Biancarelli DL, Biello KB, Childs E, Drainoni M, Salhaney P, Edeza A (2019). Strategies used by people who inject drugs to avoid stigma in healthcare settings. Drug Alcohol Depend.

[14] Giordano TP, Hartman C, Gifford AL, Backus LI, Morgan RO (2009). Predictors of retention in HIV care among a national cohort of US veterans. HIV Clin Trials.

[15] The White House. National HIV/AIDS strategy for the United States 2022–2025. Washington, D.C.; 2021. https://www.whitehouse.gov/wp-content/uploads/2021/11/National-HIV-AIDS-Strategy.pdf.

[16] National Academies of Sciences, Engineering, and Medicine. Opportunities to improve opioid use disorder and infectious disease services: Integrating responses to a dual epidemic. Washington, DC: The National Academies Press; 2020. 10.17226/25626. 32293827

[17] Springer SA, Barocas JA, Wurcel A, Nijhawan A, Thakarar K, Lynfield R (2020). Federal and state action needed to end the infectious complications of illicit drug use in the United States: IDSA and HIVMA’s advocacy agenda. J Infect Dis.

[18] Lazo M, Gange SJ, Wilson TE, Anastos K, Ostrow DG, Witt MD (2007). Patterns and predictors of changes in adherence to highly active antiretroviral therapy: longitudinal study of men and women. Clin Infect Dis.

[19] Wohl AR, Carlos JA, Tejero J, Dierst-Davies R, Daar ES, Khanlou H (2011). Barriers and unmet need for supportive services for HIV patients in care in Los Angeles County, California. AIDS Patient Care STDs.

[20] Montague BT, Kahler CW, Colby SM, McHugh RK, Squires D, Fitzgerald B (2015). Attitudes and training needs of new England HIV care and addiction treatment providers: opportunities for better integration of HIV and alcohol treatment services. Addict Disord Their Treat.

[21] Claborn K, Becker S, Operario D, Safren S, Rich JD, Ramsey S (2018). Adherence intervention for HIV-infected persons who use drugs: adaptation, open trial, and pilot randomized hybrid type 1 trial protocol. Addict Sci Clin Pract.

[22] Singer M, Clair S (2003). Syndemics and public health: Reconceptualizing disease in bio-social context. Med Anthropol Q.

[23] Consolidated criteria for reporting qualitative research (COREQ): a 32-item checklist for interviews and focus groups | The EQUATOR Network. https://www.equator-network.org/reporting-guidelines/coreq/. Accessed 6 June 2022. 10.1093/intqhc/mzm042 17872937

[24] Guest G, Macqueen KM, Namey EE. Introduction to Applied Thematic Analysis In: Applied Thematic Analysis Introduction to Applied Thematic Analysis 2014. 10.4135/9781483384436.

[25] Polydorou S, Gunderson EW, Levin FR. Training Physicians to Treat Substance Use Disorders. 2008. 10.1007/s11920-008-0064-8 PMC2741399 18803913

[26] Gray S. UMMS developing training program on medication-assisted opioid addiction treatment. UMass MedNow. 2018. https://www.umassmed.edu/news/news-archives/2018/11/umms-developing-training-program-on-medication-assisted-opioid-addiction-treatment. Accessed 9 Aug 2022.

[27] Zerbo E, Traba C, Matthew P, Chen S, Holland BK, Levounis P (2020). DATA 2000 waiver training for medical students: lessons learned from a medical school experience. Subst Abus.

[28] Scott K, Murphy CM, Yap K, Moul S, Hurley L, Becker SJ. Health professional stigma as a barrier to contingency management implementation in opioid treatment programs. Transl Issues Psychol Sci. 2020. 10.1037/tps0000245. 10.1037/tps0000245 PMC8412039 34485617

[29] Browne T, Priester MA, Clone S, Iachini A, DeHart D, Hock R (2016). Barriers and facilitators to substance use treatment in the rural south: a qualitative study. J Rural Health.

[30] Lunze K, Lioznov D, Cheng DM, Nikitin RV, Coleman SM, Bridden C (2017). HIV stigma and unhealthy alcohol use among people living with HIV in Russia. AIDS Behav.

[31] Claborn K, Becker S, Ramsey S, Rich J, Friedmann PD (2017). Mobile technology intervention to improve care coordination between HIV and substance use treatment providers: development, training, and evaluation protocol. Addict Sci Clin Pract.

[32] Nyblade L, Stockton MA, Giger K, Bond V, Ekstrand ML, Lean RM (2019). Stigma in health facilities: why it matters and how we can change it. BMC Med.

[33] Turan JM, Elafros MA, Logie CH, Banik S, Turan B, Crockett KB (2019). Challenges and opportunities in examining and addressing intersectional stigma and health. BMC Med.

[34] Frank HE, Becker-Haimes EM, Kendall PC (2020). Therapist training in evidence-based interventions for mental health: a systematic review of training approaches and outcomes. Clin Psychol Sci Pract.

[35] Beidas RS, Cross W, Dorsey S (2014). Show me, don’t tell me: behavioral rehearsal as a training and analogue fidelity tool. Cogn Behav Pract.

[36] Bearman SK, Schneiderman RL, Zoloth E (2017). Building an evidence base for effective supervision practices: an analogue experiment of supervision to increase EBT Fidelity. Adm Policy Ment Health Ment Health Serv Res.

[37] Powell BJ, Waltz TJ, Chinman MJ, Damschroder LJ, Smith JL, Matthieu MM (2015). A refined compilation of implementation strategies: results from the expert recommendations for implementing change (ERIC) project. Implement Sci.

[38] Avorn J (2017). Academic detailing “marketing” the best evidence to clinicians. JAMA.

