# Supplementary material for: Cross-training needs among community-based clinicians in HIV and substance use
Source: BMC Med Educ. 2022 Aug 19;22:629. doi: 10.1186/s12909-022-03682-3 (PMC9389712; doi:10.1186/s12909-022-03682-3)
Supplement: Supplementary file 1 — Additional file 1. HIV Provider Individual Interview Guide. [file 12909_2022_3682_MOESM1_ESM.docx]

**HIV Provider Individual Interview Guide**

**Version 6.0**

***This agenda is intended to guide data collection for the in-depth interviews with treatment providers. While the agenda is used to guide the discussion, it is not a rigid script that will be adhered to verbatim. This ensures that the facilitator gathers data on the same topics in each interview, while also allowing the flexibility to adapt and clarify questions to suit the needs of different treatment providers. The facilitator will adapt the conversation as needed according to the narrative within each interview, pursuing both the* a priori *research topics as well as any emergent relevant themes that evolve from the discussion.***

**WELCOME**

1. Thank them for coming.

2. Explain that this is a qualitative interview to help the research team understand how to improve treatment for patients with both HIV and substance use issues. We want to design a program that will be useful for both patients and treatment providers to help HIV patients with substance use issues connect to substance use treatment.

3. Sign the consent form, researchers will keep the signed copy and return a blank form to them for their records.

**PART I: EXPERIENCES WORKING WITH HPWUD**

***Intent:*** *The intent of this section is to understand clinician’s experiences in working with HIV-infected people who use drugs. We want to understand their perspectives in regards to challenges and facilitators to working with this population.*

- Tell me about the patients you generally see, their general demographics, and how alcohol and drug use/HIV fits into those demographics.
- Do you mostly work with a specific demographic (HIV/SUD)?

*Experience working with HIV/SUD:*

- - So you said you’ve been working with HIV/SUD patients for [X] number of years, tell me about your experiences.

*No experience working with HIV/SU:*

- - It looks as if you’ve had limited experience with this population. Tell me about stories you’ve heard from other providers about working with this group of patients.
  - Tell me about some of the challenges working with this population (HIV+ and use drugs). Of all the challenges you just mentioned, which one’s the hardest? Why? Next hardest? Why does that one come next?
- Talk to me about what it’s like trying to connect SU/HIV patients with SU/HIV treatment.
  - Barriers to going to SU/HIV treatment.
- A lot of patients with substance use and HIV tend to cycle in and out of treatment. Why do you think this tends to happen?
  - What do you think we could do to help people stay in care and not drop-out?
- If we could wave a magic wand, what needs to be different for your patients to get into SU/HIV treatment?
- What are some strategies providers use to get patients to stay in treatment or take their meds?

**PART II: REFERRAL SYSTEMS AND COMMUNITY RESOURCES**

***Intent:*** *The intent of this section is to understand what resources in the community clinician’s refer this population to and what experiences the clinicians have had with these community organizations. We want to understand their perspectives on the utility of these resources for patients and how patients access and utilize these resources once referred. Additionally, we want to understand how providers communicate with other health care providers located off-site. We are specifically interested in the coordination of dual care among HIV providers and SU providers. In this respect, we want to know if and how providers refer patients to SU/HIV treatment, how the providers communicate with SU/HIV providers about their patients, and what the referral process is like.*

- Tell me about some of the community resources that are available for HPWUD.
  - How do you use these resources with your patients now?
  - How helpful have these resources been to you as a clinician?
  - How accessible have these resources been for you as a provider? For your patients?
  - What can be changed to make it easier for you as a provider to use these resources? To make it easier for your patients?
- How often – if ever - do you communicate with SU/HIV providers about a patient (weekly, monthly, few times a year, never)? How about with other medical providers?

*If the person answers that they have communicated with a provider:*

- - How have you communicated with providers in the past (e.g., phone, fax, email, etc.)?
  - What works best for you when you have needed to communicate with a provider about a patient?

*If the person answers that no they have not communicated with a provider:*

- - What are the main reasons why you haven’t communicated with a provider in the past?
- How often – if ever – do you make referrals for your patients to see a SU/HIV treatment provider? (weekly, monthly, few times a year, never) How about referrals to other treatment providers?
  - What’s your typical process for making tx referrals?
  - What’s your sense of what usually happens after you make a referral?
- Tell me about some of the challenges in working with other treatment providers on a specific patient.
- Tell me about some things that would make it easier to communicate with offsite treatment providers?

**PART III: INTERVENTION COMPONENTS**

***Intent:*** *The intent of this section is to evaluate intervention content and gather specific recommendations about topics and materials to tailor the Life Steps adherence intervention and BMI to HPWUD. Specifically, we want to know: (a) what information regarding HIV and substance use will be helpful for this patient population, (b) what resources should be included, and (c) how such a program should be structured. We want the intervention to be acceptable, feasible, and relevant to both providers and the target population.*

- Think about the last time you worked with a patient who had HIV and also had a drug problem.
  - *For Physicians:* Tell me about what the appointments look like. What gets talked about? Was this patient also in SU/HIV treatment?
  - What topics did you cover with this patient? (types of things you talked about?)
  - What materials – if any - did you use in your work with this patient?
  - Where did the treatment take place? (e.g., your office, patient’s home, etc.)?
  - How long did your treatment with the patient last? Why?
- Now let’s think about what you could do if you had a new patient with HIV and a drug use problem – and you had unlimited time and resources to deliver whatever treatment you wanted.
  - What types of topics would you most want to cover in your work with this patient?
  - What topics do you think the patient would most want to cover?
    - If you had plenty of time, what other topics would you want covered that you haven’t mentioned yet?
  - What materials, if any, would you most want to use (e.g., workbook, handouts, books, mobile app, list of resources, websites, etc.)?
    - What materials, if any, do you think the patient would most want to use?
  - Where would you like the treatment to take place (e.g., at the HIV clinic/SU clinic, at home, online, on a mobile app, etc.)?
    - Where do you think the patient would most like the treatment to take place?
  - How would you most like to meet with your patients (e.g., individually, with the family, in a group)?
    - How do you think your patients would most like to meet with you?
  - Who would you prefer to have deliver information about substance use to your patients – you, a SU/HIV provider, or another provider?
  - How long do you think this type of treatment should last (e.g., one-time shot, weekly, monthly)?
- Here is a list that we’ve come up with so far.
  - Tell me what’s helpful? not helpful?
  - What is missing?

**PART IV: EVALUATION OF TRAINING PROTOCOL AND RESOURCES**

***Intent:*** *The intent of this section is to evaluate clinician preferences in regards to training in comorbid HIV and substance use issues, and training in the intervention protocol. We want to understand their current level of training in substance use and how they received this training. We want to learn what topics they would like more training in and the preferred method to receive this training. Additionally, we want to understand provider preferences in regards to supervision.*

- *Training:*
  - So, talk to me about the role you might play in this intervention that we just developed?
  - How do we help you to do your part to support your patients?
  - Tell me about any previous training you might have had in addiction and substance use issues.
  - How do you currently get formal training, if any, in substance use issues? In the future, as you work with pts with HIV/SUD, what are some gaps in your training or additional learning needs that will help you better manage these pts?
    - What specific topic areas would be most helpful for you?
    - How would you most like to receive training (e.g., online, in-person)?
    - How often would you like to receive training? (e.g., once, few times year, never)
    - How many training sessions do you think you would need to feel confident in working with patients who have HIV and drug use problems?
    - What resources would be most helpful to you to work with this population (e.g., manuals, online trainings and resources, books, etc)?
- *Supervision:*
  - How often do you get supervision?
    - Do you feel that your supervision is too often, not often enough or just right?
    - If you could make any changes to your supervision, what – if anything - would you change?

**PART V: CLOSURE**

- What concerns do you have about addressing both HIV care and alcohol/drug use with patients?
- Is there anything else that you think is important for us to know?

***Turn off recorder.***
